# Distinct effects of acute versus chronic corticosterone exposure on Zebra finch responses to West Nile virus

**DOI:** 10.1093/conphys/coz094

**Published:** 2019-12-05

**Authors:** Lynn B Martin, Meredith E Kernbach, Thomas R Unnasch

**Affiliations:** Global Health and Infectious Disease Research Center, University of South Florida, Tampa, FL 33620, USA

**Keywords:** Disease, immune, immunocompetence, stress, tolerance, wildlife, zoonosis

## Abstract

Wild animals are exposed to both short- (acute) and long-term (chronic) stressors. The glucocorticoid hormones, such as corticosterone (CORT), facilitate coping with such stressors, but these hormones can have quite distinct effects contingent on the duration of their elevation. Previously, we found that experimental elevation of CORT for 2 days (via implantation) affected zebra finch (*Taeniopygia guttata*) responses to West Nile virus (WNV). CORT-elevated birds had higher viremia for at least 2 days longer than controls, and West Nile virus (WNV)-associated mortality occurred only in CORT-elevated birds. Here, we queried how acute elevations of CORT, via injection an hour prior to WNV exposure, would affect host responses, as short-term CORT elevations can be protective in other species. Although CORT injections and implantations elevated circulating CORT to a similar degree, the type of CORT exposure had quite distinct effects on WNV responses. CORT-implanted individuals reached higher viremia and suffered more mortality to WNV than control and CORT-injected individuals. However, CORT-implanted birds maintained body mass better during infection than the other two groups. Our results further support the possibility that chronic physiological stress affects aspects of host competence and potentially community-level WNV disease dynamics.

## Teaser

Summary statement

Stress hormones affect immune responses, behaviour and other host traits that can influence how individual hosts contribute to disease cycles (i.e. competence). We found that differences in the duration of experimental elevations of one hormone, corticosterone, had very different effects on zebra finch responses to West Nile virus. Chronic elevations enabled birds to become infectious and more tolerant of WNV whereas birds experiencing acute elevations generally resembled untreated controls.

## 1. Introduction

Steroid hormones, such as corticosterone (CORT), profoundly affect vertebrate immunity and parasite-directed behaviours (Wingfield, 2003; Glaser and Kiecolt-Glaser, 2005; Dhabhar, 2009; Martin, 2009b), but how these and other hormones affect host competence is unclear (Martin and Boruta, 2014; Barron *et al.*, 2015; Martin *et al.*, 2016). Host competence represents the ability of a host to transmit a parasite to another host or vector (Keesing *et al.*, 2010; Han *et al.*, 2015b) and is an amalgamation of (i) risk of exposure to parasites, (ii) host susceptibility upon exposure, (iii) host suitability for parasite replication (including the duration of infectiousness) and (iv) the ability of an infected host to encounter and interact with another host or vector once infected (Luis *et al.*, 2013; Barron *et al.*, 2015; Gervasi *et al.*, 2015; Downs *et al.*, 2019; Martin *et al.*, 2019). The relative lack of study of CORT on host competence is somewhat surprising given that many stressors (Beldomenico and Begon, 2016), anthropogenic and natural, alter CORT regulation and are implicated in the emergence of several diseases (Martin *et al.*, 2010b; Martin and Boruta, 2014; Hassell *et al.*, 2017). Moreover, epidemiological cycles of parasites are often driven by heterogeneity in, and covariance among, individual host traits (Lloyd-Smith *et al.*, 2005; Bansal *et al.*, 2007; Lloyd-Smith *et al.*, 2009; Hawley and Altizer, 2011; Martin and Al, 2019), some of which arises because of stress (Gervasi *et al.*, 2015; Vazquez-Prokopec *et al.*, 2016). Superspreaders, for instance, are disproportionately responsible for transmitting parasites to other hosts because of their distinct behaviours affecting transmission (Lloyd-Smith *et al.*, 2005; Beldomenico and Begon, 2010; Martin and Al, 2019), but it remains unclear whether stress plays a role in superspreading. Perhaps the best-known human example is Typhoid Mary, an individual thought responsible for 53 cases of *Salmonella typhi* mortality in humans. Although the commonness of ‘Typhoid Marys’ is unknown, 20% of hosts cause 80% of many infections (Woolhouse *et al.*, 1997), highlighting the need to investigate the mechanistic basis of individual heterogeneity in competence (Keesing *et al.*, 2009; Keesing *et al.*, 2010; Han *et al.*, 2015a; Olival *et al.*, 2017).

Our interest here was to investigate directly the role of acute versus chronic CORT elevations on multiple aspects of avian competence for West Nile virus (WNV). WNV is a flavivirus, primarily transmitted among *Culex sp*. mosquitoes and passerines (Kramer *et al.*, 2007), that was introduced to New York in 1999 and reached the west coast of the USA by 2004 (LaDeau *et al.*, 2007). Based on experimental infections, hosts (species, populations and individuals) vary extensively in response to WNV (Komar *et al.*, 2003; Reisen and Hahn, 2007; Brault, 2009; Wheeler *et al.*, 2009). Although CORT can affect some immune responses to WNV, it is only just becoming clear that CORT effects can influence other aspects of competence as well (Martin *et al.*, 2016). Traditionally, chronic CORT elevations have been expected to compromise host competence, as hosts that become more susceptible or less resistant to parasites via immunosuppression might also be more likely to die because of infection (Dhabhar, 2009). On the other hand, CORT might oftentimes promote competence by altering one or more processes once the host and parasite come into contact. Specifically, CORT could augment immune defences at the time of exposure, thereby reducing susceptibility (Martin, 2009a, b), or bolster resistance over the course of an infection. CORT could also affect forms of parasite tolerance (i.e. the effects of parasites on host performance and/or fitness (Raberg *et al.*, 2007; Medzhitov *et al.*, 2012; Burgan *et al.*, 2018, 2019b). CORT might attenuate tolerance if it negatively affected the cells and molecules that hosts use to moderate parasite effects. Conversely, CORT could enhance tolerance by shifting leukocyte repertoires or actions to less immunopathological forms. Although counter intuitive, potentiation of tolerance might be relatively common given that attenuation of inflammatory responses (Gola *et al.*, 2017), including those regulating sickness behaviours (Raberg *et al.*, 2009; Sears *et al.*, 2011), often occur in response to CORT.

From previous work, we expected that enduring CORT elevations will often foster host competence. CORT alters some behaviours that enhance host exposure to feeding by mosquito vectors such as the timing or amount of movement activity (Breuner *et al.*, 1998) as well as restfulness during periods of vector foraging (Astheimer *et al.*, 1992). We also recently discovered that experimental elevation of CORT doubled the biting preference of *Culex* vectors for individual zebra finches (*Taeniopygia guttata*), even though CORT also increased defensive behaviours of the same birds (Gervasi *et al.*, 2016). We also know that CORT affects various vertebrate immune functions (Sapolsky *et al.*, 2000; Dhabhar, 2009) including the regulation of the cytokines, leukocytes and other mediators of the duration and intensity of viremia (Applegate and Beaudoin, 1970, Sternberg, 2006, Adelman and Martin, 2009). In another recent study using zebra finches, experimental CORT elevations increased the magnitude and duration of WNV viremia, although impacts on host tolerance were modest and WNV-mediated mortality occurred outside the window of transmissibility (Gervasi *et al.*, 2017b).

Here, we sought to add a new dimension to the above work, namely an investigation of the effects of different CORT exposure durations on finch responses to WNV (Adamo and Parsons, 2006; Dhabhar, 2009; Martin, 2009a). CORT tends to compromise many of the mechanisms that underlie viral susceptibility and dissemination (Martin *et al.*, 2016; Gola *et al.*, 2017) when it is chronically elevated (Sapolsky *et al.*, 2000). Over short periods, though, many antiviral and inflammatory processes in the skin and blood (Dhabhar and McEwen, 1997; Dhabhar and McEwen, 1999) are enhanced by CORT. Our goal here was to determine whether experimental acute and chronic CORT elevations affected finch responses to WNV differently (Gervasi *et al.*, 2016; Gervasi *et al.*, 2017b). In light of previous results (Gervasi *et al.*, 2016; Gervasi *et al.*, 2017b), we expected that CORT-implanted birds would be (i) more infectious (i.e. longer duration WNV viremia above the 5-log transmission threshold), (ii) less tolerant (i.e. less able to maintain body mass during infections) and (iii) more likely to succumb to WNV infection than controls (Gervasi *et al.*, 2016; Gervasi *et al.*, 2017b). By contrast, we expected CORT-injection to bolster resistance such that infectiousness and tolerance would be immeasurable and WNV-associated mortality negligible. CORT-injected birds were intended to simulate individuals having experienced a recent, transient stressor (e.g. a storm, a failed predation attempt, or an encounter with a competitor). CORT-implanted birds, on the other hand, were intended to simulate individuals having experienced a short, chronic stressor (e.g. prolonged bad weather or food shortage).

## 2. Methods

### 2.1. Animal husbandry and CORT and WNV exposure

We obtained adult male and female zebra finches (*Taeniopygia guttata*) from an active breeding colony maintained at the University of South Florida. Finches were housed in 15–18 different free-flight cages (90 × 60 × 60 cm) prior to the experiment in separate sex groups. Birds selected for the study were randomly chosen from these groups, and sexes equally distributed among treatments. During the experiment itself, birds were housed alone in conventional songbird-sized cages (30 × 30 × 30 cm), but in audial and visual contact of each other. As before (Gervasi *et al.*, 2017a, b), finches were acclimated to new housing conditions for 3 days prior to hormone implantation surgeries and were allowed to recover from surgery for 2 days. They were then moved to the University of South Florida (USF) Animal Biosafety Level (ABSL) 3 facility where they acclimated for another 24 h before being exposed to WNV or CORT/vehicle injections. For the duration of the study, all birds were fed ABBA 1900 exotic finch food (ABBA Products Corp., Hillside, NJ). The photoperiod was kept at 13 h light:11 h dark (on at 0600 and off at 1900), and room temperature and relative humidity were ~ 21°C and ~ 50%, respectively. All procedures complied with approved USF animal care and use committee and biosafety protocols.

**Figure 1:**
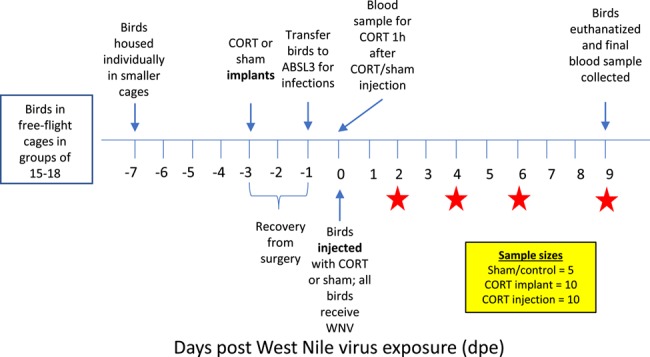
Schematic of study design used here including the timing of implanting/injecting CORT or sham treatments, WNV exposure and blood sampling for viremia. Stars denote time points of blood collection for WNV viremia.

Figure 1 is provided to summarize our experimental design visually. For chronic CORT treatments, we implanted 25 adult zebra finches subcutaneously (s.q.) with either 1 or 2 CORT-filled or 1 or 2 empty silastic tubules according to previously published protocols (*n* = 10 CORT (five birds with two and five birds with one filled implant) and *n* = 15 with one empty implant (Ouyang *et al.*, 2013; Gervasi *et al.*, 2016; Gervasi *et al.*, 2017b). This effort to implant different numbers of tubules for CORT birds was intended to reveal CORT concentrations at which protective and detrimental effects occurred. As preliminary analysis indicated no statistically significant effects of number of implants on any traits, treatments were collapsed into single groups (i.e. CORT implant or sham). All implants were sealed (Dow Corning, Midland, MI, product #732) days prior to implant, but just before implantation, a 0.5-mm hole was made through both sides of each implant, which previous work revealed helps the efflux of hormone (Ouyang *et al.*, 2013). All implants were administered on the flank, while birds were sedated with light isoflurane anaesthesia as assessed by lack of responsiveness to toe-pinches. After implantation, surgical adhesive (Vetbond, 3 M, St. Paul, MN, product #1469) was used to seal wounds. All birds returned to normal activity (perching and feeding) within ~ 5 minutes.

Two days later, birds were moved to the ABSL-3 facility and injected s.q. with either CORT (*n* = 10; 2 μl of either a 1600-μg ml^−1^ (high) or 80-μg ml^−1^ (low) solution; five individuals of each dose) or peanut oil vehicle (*n* = 20). One hour later, all birds were exposed to 3 × 10^8^ PFU WNV (NY99) via s.q. injection (Gervasi *et al.*, 2017a, b). As preliminary analysis also indicated no difference between injection concentrations, all injected birds were collapsed into one ‘injected’ group. Altogether, our approach meant that experimental birds were exposed to elevated CORT for either 2d (implant group, *n* = 10) or approximately 1 h (inject group, *n* = 10), whereas controls received no CORT exposure of either form but sham forms of both injections and implants (*n* = 5). Blood samples (to quantify viremia; 70 μl) were then collected at 0, 2, 4, 6 and 9 days post-exposure (d.p.e.), serum was removed from blood samples and stored at −40°C until RNA extraction. Viremia was not measured in day 0 samples; this material was only used to quantify circulating CORT.

To maximize the chance that we quantified CORT in response to manipulations, not disturbance by investigators, blood samples were collected from each bird 1 h after CORT/vehicle injections but within 3 minutes of a researcher contacting the cage in which an individual bird was housed, just prior to WNV exposures. Each day of blood sampling, we also recorded body mass of each bird, and each day of the post-WNV-exposure period, we recorded whether an individual had died since the last observation. In the end, three treatment groups were available for comparison: (i) controls (sham implanted and vehicle injected), (ii) CORT-implanted (hormone filled-silastic implanted and vehicle injected) and (iii) CORT-injected (empty-silastic implanted and CORT-in-vehicle injected).

### 2.2. CORT concentrations and WNV quantification

We measured CORT concentrations using enzyme immunoassay (EIA) kits (Arbor Assays, Ann Arbor, MI, product # K017-H), which were previously validated for this species (Martin, unpublished data). Samples were measured in duplicate and concentrations extracted from a standard curve according to kit instructions. We used quantitative PCR to measure WNV viremia, an approach developed and validated in prior studies in our group (Gervasi *et al.*, 2017a, b; Burgan *et al.*, 2018). Briefly, viral RNA was extracted from serum samples with the QIAmp Viral RNA kit (Qiagen Cat. No. 52906). We used 10 μl of serum diluted in 130-μl sterile PBS and followed the kit protocol for all steps. We then used a one-step Taq-based kit to quantify viral RNA in samples (iTaq Universal Probes One-Step Kit; Bio-Rad Cat. No. 1725141). As in the past, our forward primer sequence was 5’ CAGACCACGCTACGGCG 3′; reverse sequence was 5’ CTAGGGCCGCGTGGG 3′; and our WNV probe sequence was 5′ [6~FAM] CTGCGGAGAGTGCAGTCTGCGAT [BHQ1a~6FAM]. All samples were measured in triplicate, and a negative control and a known WNV-positive control were run concurrently with experimental samples on all plates. Standard curves were generated from serial dilutions of the same stock virus used in finch inoculations, in which viral titre was quantified previously using Vero cell plaque assay. All samples on all plates were captured by our standard curves.

### 2.3. Data analysis

We used one-way ANOVA followed by simultaneous Bonferroni *post hoc* tests to discern whether CORT differed among treatment groups. We then used linear mixed models to ascertain how CORT treatment affected (i) resistance (i.e. viremia over the 9 days after WNV exposure) and (ii) tolerance (i.e. relationship between body mass and WNV viremia throughout the infection). For the former, we included CORT treatment, days post-WNV-exposure (dpe) and their interaction as fixed effects and individual bird identity as a random effect (to account for repeated measures) to attempt to predict log WNV viremia. WNV viremia (log_10_) was normally distributed. To evaluate CORT effects on tolerance, we treated body mass as the dependent variable (also normally distributed) and included log_10_ WNV viremia, CORT-treatment and the interaction of treatment and viremia as fixed effects; again, individual bird was treated as a random effect, and body mass at the time of WNV exposure was included as a covariate to account for pre-existing differences in vigour among individuals (Gervasi *et al.*, 2017a; Burgan *et al.*, 2018). In essence, tolerance represents the relationships between WNV burden and performance (Raberg *et al.*, 2007), and in this study, we used defence of body mass as a measure of performance, as others were difficult to quantify given that birds were infected with WNV. To estimate individual tolerance, one can either estimate residual variation in performance from the population mean relationship with burden, or one can estimate the slope of burden/performance relationships at the individual level (Raberg *et al.*, 2009); these alternatives are highly correlated (Burgan *et al.*, 2019a). We estimated two forms of tolerance, both using defence of body mass as our proxy of performance (Raberg *et al.*, 2007). First, we analyzed all body mass and viremia data for all individuals at all time points, as CORT effects on tolerance could lag and/or accumulate over the course of infection. Second, we analyzed only data from days 4 and 6 post-WNV-exposure, as this period is the one in which viremia tends to supersede the transmission threshold, making transmission to vectors possible (Turell *et al.*, 2000). We report betas (regression slopes +/− 1 standard error) for all significant interactions between WNV and body mass change, as these values connote the magnitude effect of viremia on body mass across the measured period. Finally, we used a Cox regression model to determine whether CORT treatment affected WNV-associated mortality. In this model, only CORT treatment was included as a predictor, and we used Wald tests and estimated hazard ratios to compare mortality rates among treatment groups. We used SPSS v24 for all analyses, and GraphPad Prism v6 for all figures, setting alpha to 0.05.

## 3. Results

### 3.1. CORT concentrations

**Figure 2:**
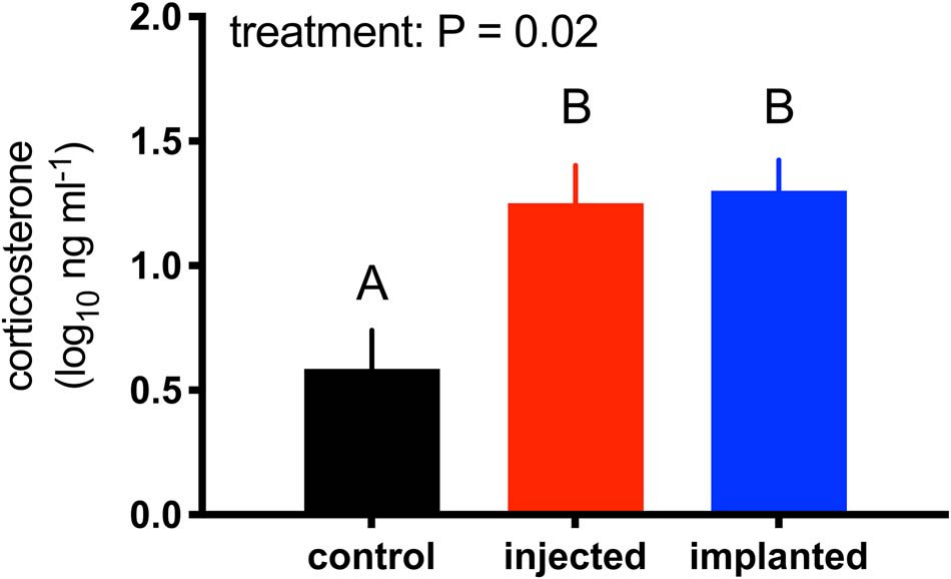
Corticosterone injection and implantation elevated circulating corticosterone to similar degrees, in zebra finches to controls, 1 h prior to WNV inoculation. Bars depict means +1SE and letters denote group membership by Bonferroni *post hoc* comparisons.

Manipulations effectively altered circulating CORT (F_2,19_ = 5.43, *P* = 0.02). Birds in both the injected and implanted groups had higher CORT concentrations than birds in the sham group but comparable concentrations to each other (Fig. 2). As noted above, these samples represent circulating CORT concentrations 2d after CORT implantation or ~ 1 h after CORT injection, depending on the treatment group to which a bird was assigned.

### 3.2. WNV resistance

**Figure 3:**
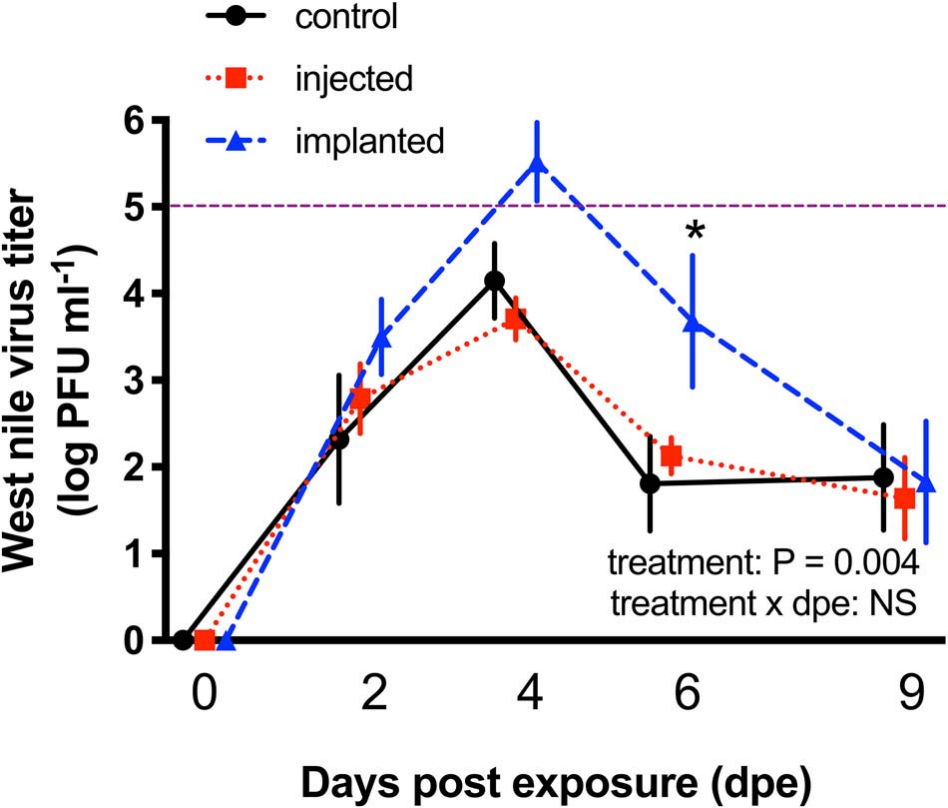
Corticosterone effects on West Nile virus viremia in zebra finches: sham (black lines and symbols), injected CORT (red lines and symbols) and implanted CORT (blue lines and symbols). Purple horizontal line denotes threshold that titre must surpass to be transmissible to vectors. Bars depict means +/− 1SE.

CORT treatment and time, but not their interaction (F_8,91_ = 1.48, *P* = 0.18), predicted WNV viremia (Fig. 3). Titres increased and eventually decreased over the course of the measurement period, exhibiting kinetics comparable to other studies (F_4,91_ = 40.13, *P* < 0.001). Across the entirety of the infection, birds in the implanted CORT group exhibited higher viremia than birds in the other two groups (F_2,91_ = 5.89, *P* = 0.004). Only on day 6, however, did we detect a difference among groups based on simultaneous *post hoc* (Bonferroni) comparisons (asterisk in Fig. 3).

### 3.3. WNV tolerance

**Figure 4:**
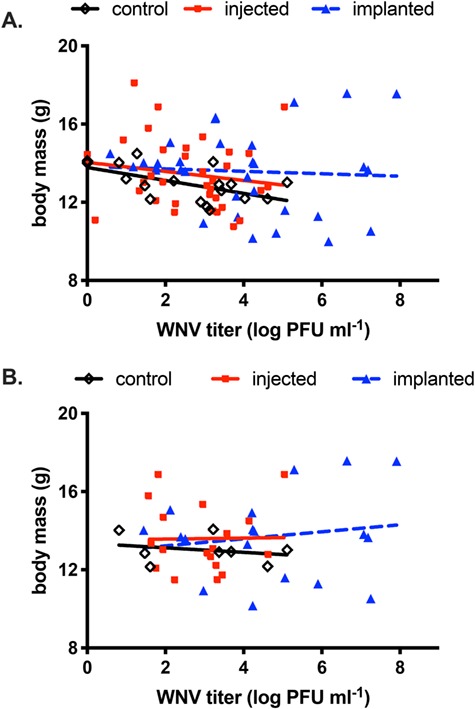
Corticosterone effects on WNV tolerance in zebra finches. A. Across the entire duration of the infection, body mass declined with increasing WNV titre in control (open diamond symbols) and CORT-injected (solid square symbols) birds, but not CORT-implanted birds (solid triangle symbols). Dashed regression line for implanted birds depicts non-significant trendline. B. On days 4 and 6 only of infection, CORT-injected birds had lower tolerance than CORT-implanted birds, whereas controls were intermediate of both. Dashed regression line for implanted birds depicts non-significant trendline.

Controlling for vigour (body mass prior to WNV exposure; F_1,78_ = 324.5, *P* < 0.001), CORT treatment (F_2,78_ = 3.4, *P* = 0.04) predicted body mass over the course of entire infection, but the effects of WNV titre on body mass differed among treatments (CORT treatment x WNV viremia: F_2,78_ = 5.00, *P* = 0.009). There was no detectable main effect of viremia on body mass though (F_1,78_ = 0.36, *P* = 0.55). Relative to the implanted group, body mass decreased with viremia in the injected ($\upbeta$ = −0.39 +/−0.13, t_78_ = −2.98, *P* = 0.04) group but not the control group ($\upbeta$ = −0.26 +/−0.15, t_78_ = −1.84, P = 0.07; Fig. 4A). As viremia effects on body mass in the CORT-implanted group were not statistically significant, we do not report a beta. Altogether, these results indicate that CORT-injected and control birds tolerated WNV similarly, whereas CORT-implanted birds might have tolerated WNV better, though again the viremia-body mass relationship was non-significant in this group. When CORT treatment and viremia effects on body mass were investigated on only days 4 and 6 post-exposure (i.e. when transmission to vectors would have been possible because at least some individuals would have had viremia above the transmission threshold), WNV titre alone did not predict body mass (F_1,37_ = 0.10, *P* = 0.75), but both CORT treatment (F_2,37_ = 4.19, *P* = 0.02) and the treatment by WNV titre interaction (F_2,37_ = 4.85, *P* = 0.01) did. Vigour, too, continued to be a significant predictor of body mass change (F_1,37_ = 229.67, *P* < 0.001). As above for the entire course of infection, the control group ($\upbeta$ = −0.59 +/−0.19, t_37_ = −3.07, P = 0.04) tolerated WNV less than the CORT-implanted group, but tolerance in the control group was indistinguishable from the injected group ($\upbeta$ = −0.22 +/−0.20, t_37_ = −1.13, *P* = 0.64; Fig. 4B).

### 3.4. West Nile-associated mortality

**Figure 5:**
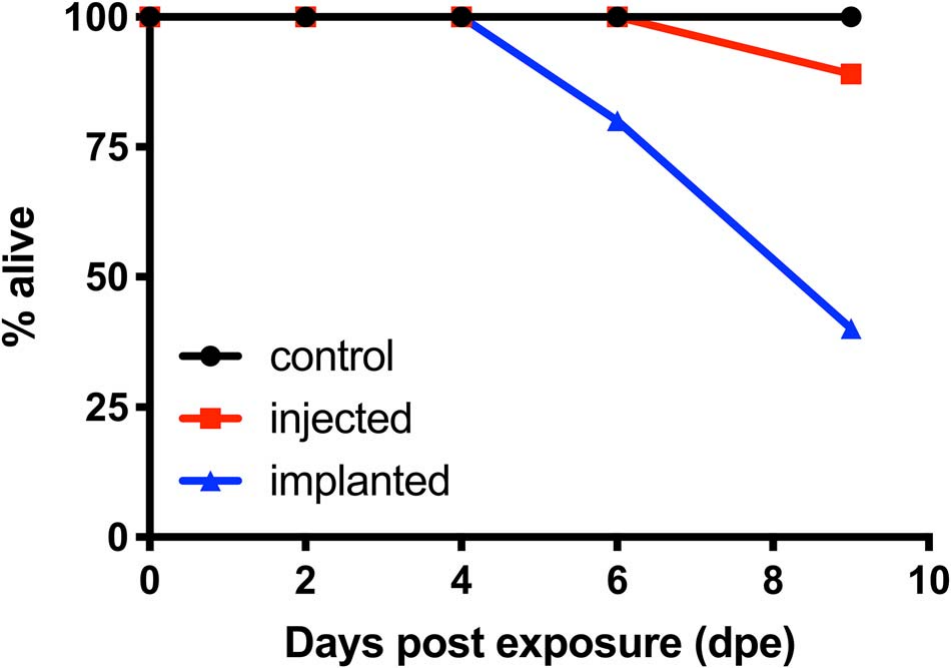
Effects of corticosterone on mortality risk to West Nile virus in zebra finches.

CORT treatment affected mortality post-exposure to WNV (χ_2_^2^ = 9.48, *P* = 0.01): no mortality was observed among controls, and modest mortality was observed among injected birds, but ~ 40% of implanted birds died by day 6 post-exposure to WNV (Fig. 5). Relative to controls, there was no difference in mortality rate for injected birds (estimated hazard ratio (HR) = −13.5 +/−414.2, *P* = 0.97), but implanted birds died at about twice the rate of controls (HR = −2.13, *P* = 0.05).

## 4. Discussion

CORT injections were not protective against WNV infections in zebra finches, as we predicted. However, as we saw in a previous study (Gervasi *et al.*, 2017a), CORT implantations elevated viremia, minimally affected tolerance and led to modest mortality but mostly outside the window of infectiousness to vectors. In other words, only CORT-implanted birds ever became competent for WNV; CORT-injected birds resembled sham controls in most ways except elevated CORT at the time of sampling. These distinct effects of CORT on aspects of competence were observed in spite of similar circulating hormone concentrations in the two CORT-treated groups. Although we do not think these single CORT measures capture the manifold complex effects likely to have arisen (and differed) between implanted and injected birds, they tell us that at least some time during the study, CORT-injected birds reached the same CORT concentrations as the implanted individuals. Below, we interpret these results in the context of health of individual birds and disease dynamics at the community-level (Acevedo-Whitehouse and Duffus, 2009).

### 4.1. Effects of CORT on WNV resistance

CORT is well known to change the immune system and various behaviours of hosts (Avitsur *et al.*, 2006), but how such effects combine to impact host competence is less clear (Ben-Nathan, 2013; Beldomenico and Begon, 2016). Relevant experimental studies in wildlife are fairly rare (Mclean, 1982; Thomas and Woodley, 2015; Fonner *et al.*, 2017) and mostly limited to amphibians. Those studies tend to focus on exposure to *Batrachochytrium dendrobatidis* (*Bd*), the causative agent of chytridiomycosis, a disease that caused many declines and extinctions of frog and toad populations (Longcore *et al.*, 1999). Presently, there is no consensus about the effects of CORT on host resistance to *Bd*, perhaps because of methodological differences among studies (Gabor *et al.*, 2015). For instance, in American toads (*Anaxyrus americanus*), two different CORT doses increased resistance to *Bd* (Murone *et al.*, 2016), but a similar study on two life stages of two other frog species found no effect of CORT on resistance. In this latter case, *Bd* burden was quite low throughout the study, so any CORT impacts might have been masked by a floor effect (Searle *et al.*, 2014).

In terms of CORT effects on WNV resistance, only three studies have involved experimental CORT manipulations (besides the zebra finch work discussed above). The first involved domestic dogs in which treatment with a high dose of synthetic CORT led to higher WNV viremia but no greater morbidity than controls (Bowen *et al.*, 2006). These findings have little relevance for natural WNV dynamics because viremia in dogs never reaches levels that biting vectors can become infectious themselves. Another example involving domesticated chickens *(Gallus gallus)* revealed that CORT treatment elevated and extended viremia relative to controls. Although, in principle, viremia in chickens can surpass the transmissible threshold for vectors, viremia never reached such levels in this study (Jankowski *et al.*, 2010). A final example, involving a similar hormone-implant paradigm in Northern Cardinals (*Cardinalis cardinalis*) to the one we used here, revealed effects of CORT on aspects of competence for this key species for WNV transmission (Kilpatrick *et al.*, 2007). There, though, CORT-implanted cardinals died at a much higher rate than controls, even though viremia never differed between treated and control birds (Owen *et al.*, 2012) but well exceeded the transmission threshold.

Unfortunately, in the above and likely most studies of stress and competence, the conditions of the experiment could bias outcomes. Captivity is well known to affect the immune responses of wild birds (Buehler *et al.*, 2008; Martin *et al.*, 2011; Gao *et al.*, 2017; Love *et al.*, 2017), so in the above cardinal study (and perhaps those on some amphibians), resistance, infection-induced mortality or both, might have been affected by the psychological adversity individuals experienced when housed in unfamiliar conditions. Our reliance on domesticated zebra finches too might give a naturally unrepresentative impression of how CORT affects competence in wildlife. This host species is Australian, and West Nile virus arrived to Australia well after zebra finches became common as a model organism (Warren *et al.*, 2010). We originally chose to study zebra finches so as to avoid the potentially confounding effects of stress, co-infection, prior exposure or various other issues that could occur for wild animals held in captivity. Also, just as model organisms, from rodents to fruit flies, have been informative for the improvement of human health, investigations of immune responses in domesticated birds should be useful to understanding stress effects on aspects of competence in wildlife. True, domestication can influence immunity and other defences against parasites (Trut *et al.*, 2009; Christie *et al.*, 2016), as can *ad libitum* food, stable, hospitable climates and other unnatural artefacts of captivity. However, abiotic and biotic factors are also spatiotemporally heterogenous in the wild, so it is unclear just how much food should be restricted or temperature or other factors altered to represent natural variation. Most importantly, the experimental form of our work makes it easier to attribute causality to CORT; we directly manipulated it and held all else constant. Clearly, additional research on the effects of CORT on WNV competence in diverse avian species will be valuable, and caution should be used when extrapolating our results to other wildlife.

### 4.2. Effects of CORT on WNV tolerance

In spite of the challenges inherent to studying wild birds in captivity or studying domesticated species as surrogates for free-living hosts, it was intriguing to observe some supportive effects of chronically elevated CORT on one form of WNV tolerance. Both CORT-injected and control finches tended to lose body mass when viremia reached its highest levels (Fig. 3). However, many CORT-implanted birds maintained their body masses under the same conditions, although many died from infection towards the end of the study. Similar results, in terms of mortality, were observed in Northern Cardinals (Owen *et al.*, 2012), but in those birds, CORT implants did not enhance viremia. In other zebra finches, we found some evidence that chronic CORT elevations tended to reduce tolerance, at least until individuals succumbed to their high viral burdens. These results hint that for a short time, chronically stressed birds might impose a high transmission risk, particularly if they are also conspicuous to vectors; at the same time, they are most infectious (Gervasi *et al.*, 2016). However, the small sample sizes in this study as well as the relatively few studies of avian WNV tolerance and CORT in general make us reticent to make strong conclusions.

The ecological salience of our findings warrants more investigation, though, as the consequences of CORT on tolerance in the form of body mass defence are apt to be complex. Many traits capture information about how an individual host copes with an infection, but few capture the manner by which such effects influence host competence (Burgan *et al.*, 2019a; Martin *et al.*, 2019). Body mass changes in response to viral infection are obscure in this sense, as they can be related to fitness and health in songbirds, but rarely are such links simple (Brown, 1996). Over short time scales, such as in the present study, the degree of mass loss observed can be due solely to water balance. Dehydration might have little consequence for transmission of virus to vectors. Moreover, in captivity, compensation for mass loss is comparatively simple by increasing food intake. Subsequently, CORT might potentiate WNV competence in some songbirds, at least in the sense that individuals might remain viable and active for short periods before they die from infection. However, supportive effects of CORT on tolerance might not occur in nature if birds are unable to find food or if dehydration is not the sole reason for mass loss. There is also the likelihood that host species will differ in how they behave during infections (Budischak and Cressler, 2018; Hite and Cressler, 2019). For instance, house sparrows (*Passer domesticus*) *gain* body mass when infected with viruses (Coon *et al.*, 2011; Burgan *et al.*, 2018; Kernbach *et al.*, 2018) and related stimuli (Martin *et al.*, 2010a). Yet other research indicates that some individuals in multiple songbird species continue to perform well until just hours prior to death (Gervasi *et al.*, 2017a; Burgan *et al.*, 2018; Kernbach *et al.*, 2018). Our findings are also consistent with some (Fonner *et al.*, 2017), but not all (Murone *et al.*, 2016), work on *Bd* tolerance in amphibians. Any future efforts to resolve the effects of CORT on competence via tolerance should consider these complexities.

### 4.3. Relevance to conservation

It is impossible to study WNV competence in free-living wild birds using experimental infections, so we must rely on a blend of descriptive fieldwork and research on domesticated and wild species under controlled conditions. Only through this multi-tiered approach can biohazard risk can be minimized and ecological inference also occur. In this light, we think our work contributes some needed perspective to stress and competence in wildlife (Acevedo-Whitehouse and Duffus, 2009; Martin *et al.*, 2010b), namely that chronic stressors might enhance competence of some hosts for short periods by damping resistance and/or fostering tolerance. Stress is often thought to affect disease in wildlife by compromising individual resistance and expediting mortality. In this scenario, infected animals would die so quickly that mortality would outpace transmission. Our work suggests, though, that effects could be subtler, and that stressed individuals could become *more* infectious for several days before death, thus exacerbating risk in some contexts. When coupled with the increased attractiveness to vectors that stress can induce (Gervasi *et al.*, 2016), such increases in infectiousness could escalate further.

Of course, the above framework implies that experimental CORT treatments equate to stress, which is clearly a simplification (Romero *et al.*, 2015). We and others agree that CORT can be manipulated in such ways as to emulate experience of natural stressors; however, the reverse is more challenging. In other words, it is very difficult to link variation in CORT in wild animals to variation in competence or its components (Angelier and Wingfield, 2013; Dantzer *et al.*, 2014). CORT regulation is exceedingly complex, which probably explains why its use as a proxy for conservation or management prioritization has succeeded in only a handful of cases (Sorenson *et al.*, 2017; Lea *et al.*, 2018). We do expect that careful characterization of CORT regulation (i.e. assessments of negative and positive feedback of this hormone) could be useful for conservation (Romero and Wikelski, 2001; Tarlow and Blumstein, 2007), though, but this outcome was not the focus of our work. Indeed, we hoped here to learn whether acute stressors, simulated pharmacologically, could be protective against WNV for individuals and communities. Our results indicate either that acute CORT pulses genuinely are not protective or that we needed to elevate CORT a little more or for a little longer. We encourage follow-up work on this topic, as well as additional research to discern at which time and by what mechanisms protective effects of CORT on avian WNV responses start to become detrimental.

## 5. Conclusion

Going forward, we advocate that much more attention be given to the role of CORT in avian resistance, tolerance and competence to WNV as well as other zoonotic and non-zoonotic infections. Several have argued that the balance of resistance and tolerance among individuals and species will have critical impacts on disease dynamics (Martin *et al.*, 2016; Adelman and Hawley, 2017; Burgan *et al.*, 2019b; Martin and Al, 2019), which are often impacted by exposure to stressors (Paull *et al.*, 2011; Beldomenico and Begon, 2016). Although our work provides no evidence that short-term CORT elevations foster protection, it and especially our prior work indicate that CORT might increase community-level risk of exposure for this multi-host infection. Given the challenges of conducting relevant research in the wild, the inherent confounds associated with studying stress effects in captivity, and the likely diverse responses that various host populations have with evolutionarily familiar and unfamiliar parasites, we caution over-interpretation of our results. We found some modest evidence that chronic CORT elevations increased WNV-potentiated tolerance, but there will be a great value in asking whether our results are representative or exceptional of other systems (Acevedo-Whitehouse and Duffus, 2009; Beldomenico and Begon, 2010; Beldomenico and Begon, 2016).
